# Serine racemase deletion attenuates neurodegeneration and microvascular damage in diabetic retinopathy

**DOI:** 10.1371/journal.pone.0190864

**Published:** 2018-01-05

**Authors:** Hironori Ozaki, Ran Inoue, Takako Matsushima, Masakiyo Sasahara, Atsushi Hayashi, Hisashi Mori

**Affiliations:** 1 Department of Molecular Neuroscience, Graduate School of Innovative Life Science, University of Toyama, Toyama, Japan; 2 Department of Molecular Neuroscience, Graduate School of Medicine and Pharmaceutical Sciences, University of Toyama, Toyama, Japan; 3 Department of Pathology, Graduate School of Medicine and Pharmaceutical Sciences, University of Toyama, Toyama, Japan; 4 Department of Pathology, Graduate School of Innovative Life Science, University of Toyama, Toyama, Japan; 5 Department of Ophthalmology, Graduate School of Medicine and Pharmaceutical Sciences, University of Toyama, Toyama, Japan; University of Florida, UNITED STATES

## Abstract

Diabetic retinopathy (DR) is a leading cause of blindness. DR is recognized as a microvascular disease and inner retinal neurodegeneration. In the course of retinal neurodegeneration, *N*-methyl-D-aspartate receptor (NMDAR)-mediated excitotoxicity is involved. Full activation of NMDAR requires binding of agonist glutamate and coagonist glycine or D-serine. D-Serine is produced from L-serine by serine racemase (SRR) and contributes to retinal neurodegeneration in rodent models of DR. However, the involvement of SRR in both neurodegeneration and microvascular damage in DR remains unclear. Here, we established diabetic model of SRR knockout (SRR-KO) and control wild-type (WT) mice by streptozotocin injection. Six months after the onset of diabetes, the number of survived retinal ganglion cells was higher in SRR-KO mice than that of WT mice. The reduction of thickness of inner retinal layer (IRL) was attenuated in SRR-KO mice than that of WT mice. Moreover, the number of damaged acellular capillaries was lower in SRR-KO mice than that of WT mice. Our results suggest the suppression of SRR activity may have protective effects in DR.

## Introduction

Diabetic retinopathy (DR) is a sight-threatening complication of diabetes mellitus that results from damage to the blood vessels of the retina. DR involves microvascular changes, such as blood-retinal barrier breakdown and death of endothelial cells and pericytes [1]. Abnormalities in the retinal vessels including basement membrane thickening and pericyte loss are early signs of the development of DR in human [2]. Furthermore, acellular capillaries, which have empty basement membrane sleeves, are caused by the damage to the vessels due to obliteration of retinal microvessels [2]. The apparance of acellular capillaries is reported as an irreversible result of diabetes-induced endothelial cell apoptosis [3]. DR is not only a microvascular disease but also a neurodegenerative disease. Rodent models have been used to investigate the mechanisms of retinal cell damage in diabetes. The streptozotocin (STZ)-induced diabetes resulted in increase in neural cell apoptosis in the retina and reduction in the thickness of the inner retinal layer as a consequence of diabetes [4, 5].

Glutamate is the major excitatory amino acid which plays an important role as a neurotransmitter in the mammalian central nervous system [6]. Functions of glutamate are mediated by glutamate receptors. Glutamate excitotoxicity has been proposed to contribute to the death of retinal ganglion cells (RGCs) in glaucoma and DR [7, 8]. Glutamate excitotoxicity on RGCs are predominantly mediated by the overstimulation of *N*-methyl-D-aspartate receptor (NMDAR) [9]. NMDARs are composed of glycine or D-serine-binding subunit (GluN1, GluN3) and glutamate-binding subunit (GluN2) families. Their expression pattern in the central nervous system is changed during development [10]. In the adult retina, GluN1 and GluN2(A-D) subunits are detected [9, 11]. D-Serine acts as an endogenous co-agonist for the glycine-binding site of the NMDAR [12]. In the retina, D-serine is present in the inner retina and serves as an endogenous co-agonist of NMDAR [13]. The synthesis of D-serine from L-serine is catalyzed by serine racemase (SRR) [14]. We reported previously that the genetic disruption of SRR (SRR-KO) attenuates NMDA-mediated excitotoxicity in the forebrain [15]. SRR also has been detected in retina [16], and expression of SRR is increased in DR rat retina [17]. Recently, it also has been reported that SRR mutation in mouse attenuates the NMDAR-mediated acute excitotoxicity in the retina [18]. However, whether SRR is involved in both neurodegeneration and microvascular damage in long-term DR remains unclear.

In this study, we examined the expression of SRR in mouse retina, and compared the degeneration of retinal neuronal cells and retinal microvascular damage between diabetic wild type (WT) and SRR-KO mice in long-term diabetes. Our results showed the attenuation of degeneration of retinal neuronal cells and microvascular damage induced by diabetes in SRR-KO retinas.

## Materials and methods

### Induction of experimental diabetes in mice

SRR-KO mice with 100% C57BL/6 genetic background were generated as previously described [19]. Animal care and experimental protocols were carried out basically in accordance with“Guidelines for the Care and Use of Laboratory Animals, DHEW, publication no. (NIH) 80–23, revised 1996” and approved by the Experimental Animal Committee of the University of Toyama (Authorization No.2015-MED-61). Diabetes was induced at eleven week-old C57BL/6 WT mice and SRR-KO mice. Mice were received intraperitoneal injections of 60 mg/kg STZ (Sigma-Aldrich, St.Louis, MO) dissolved in sodium citrate buffer (0.05 M, pH 4.5) on three successive days. After STZ injection, mice were weighed and measured blood glucose levels with a glucometer (STAT STRIP XPRESS; Nova biomedical, Japan). Mice with blood glucose levels higher than 300 mg/dl at 1 week after the STZ injection were considered to be diabetic. Mice were weighed for 24 weeks post-STZ treatment. Age-matched, non-treated WT and SRR-KO mice were used as the control. Mice were sacrificed after 6 months from the onset of diabetes.

### Western blot analysis

Non-treated control and STZ-injected WT and SRR-KO mice were anesthetized with pentobarbital sodium (100 mg/kg body weight) by intraperitoneal injection and perfused with phosphate buffered saline (PBS, pH 7.4). Eyes were enucleated and retinas were collected. Retinas were homogenized in mammalian protein extraction reagent (Pierce, Rockford, IL). Protein concentration was measured by using a Bicinchoninic acid (BCA) Protein Assay kit (Thermo Scientific, USA). Extracted protein was subjected to SDS-PAGE and transferred onto polyvinylidene difluoride membrane. The membranes were blocked with 5% skim milk in PBS containing with 0.1% Tween 20 (PBS-T) and then incubated with rabbit polyclonal anti-SRR (1:500, [20]) or anti-actin antibodies (1:2000, Santa Cruz, CA) for overnight at 4°C. After washing in PBS-T for three times, the membranes were incubated with HRP-conjugated goat anti-rabbit IgG antibody (1:5000, Invitrogen, Carlsbad, CA) for 1 h at room temperature (RT). Protein bands were detected using an ECL chemiluminescence detection system and Image Quant LAS-4000 Mini (GE Healthcare, Uppsala, Sweden).

### Immunofluorescence staining of mouse retina

For the preparation of frozen sections, the enucleated eyes were fixed in 0.1 M phosphate buffer (PB, pH 7.4) containing 4% paraformaldehyde (PFA) for overnight at 4°C and dipped in 0.1M PB containing 30% sucrose for 36 h at 4°C. Eyes were placed into the cryomold which was filled with tissue freezing compound (OCT, Sakura Finetek, USA) and were frozen in dry ice. Cryosections (20 μm) including a full length of retina passing through the optic nerve were prepared using a freezing microtome. For SRR immunostaining, sections of retina were washed in PBS, and blocked with Protein Block Serum-Free (DakoCytomation, Carpinteria, CA) for 10 min, and incubated with rabbit polyclonal anti-SRR antibody (1:200) [**20]**, diluted in PBS containing 0.1% Triton X-100 for overnight at 4°C. On the following day, the samples were washed three times in PBS and incubated with a secondary antibody, Alexa-Fluor-488-conjugated donkey anti-rabbit IgG (1:500, Invitrogen, Carlsbad, CA) for 1 h at RT. The sections were washed in PBS, and stained with DRAQ5 (Thermo Scientific, USA) for nuclear counterstain. After washing in PBS, the sections were coverslipped.

For the preparation of flatmount retinas, eyes were fixed in PBS containing 4% PFA for 30 min and were dissected as flattened wholemounts by making four radial cuts, then permeabilized in PBS containing 0.1% Triton-X for 30 min. After blocking with the Protein Block Serum Free, the retinas were incubated with goat anti-Brn3 antibody (1:200, Santa Cruz, CA) diluted in PBS containing 1% bovine serum albumin (BSA) or rabbit anti-collagen IV antibody (1:200, Chemicon, USA) and isolectin B_4_ (1:100, Enzo Life Sciences, Switzerland) diluted in PBS containing 0.1% horse serum overnight at 4 °C. After washing three times in PBS, retinas were incubated with Alexa-Fluor-647 conjugated species-specific secondary antibodies (1:500, Invitrogen, Carlsbad, CA) for 2 h at RT. The retinas were then washed in PBS, flattened and mounted on a glass slide, and coverslipped. Images were taken using a confocal laser scanning microscope (Leica TCS-SP5, Leica Microsystems, Mannhein, Germany). The number of Brn3 immunopositive cells in posterior and peripheral area of each retina images were quantified using MetaMorph software (Universal Imaging Corp, West Chester, PA). Acellular capillaries were visualized by continuance of collagen IV positive and isolectin-B4 negative. Fifteen images per retina were analyzed to count acellular capillaries. All measurements were conducted in a mouse genotype-blind manner.

### Hematoxylin and eosin staining in eyeball cryosections

Cryosections of retinas were prepared as described above and stained with hematoxylin and eosin (H&E). Images of H&E-stained sections were taken with Keyence BZ-X700 microscope (Keyence, Osaka, Japan). The thickness of the inner retinal layer (IRL) was measured at a distance of 0.5 to 1.0 mm from optic disc.

### Statistical analysis

Statistical analyses were performed using two-tailed Student's *t*-test for comparison of two groups or one-way ANOVA followed by Turkey's post hoc test for comparison of multiple groups. All results are expressed as mean ± S. E. M. Values of *p* < 0.05 were considered to be statistically significant.

## Results

### Establishment of DR mouse model

**Fig 1:**
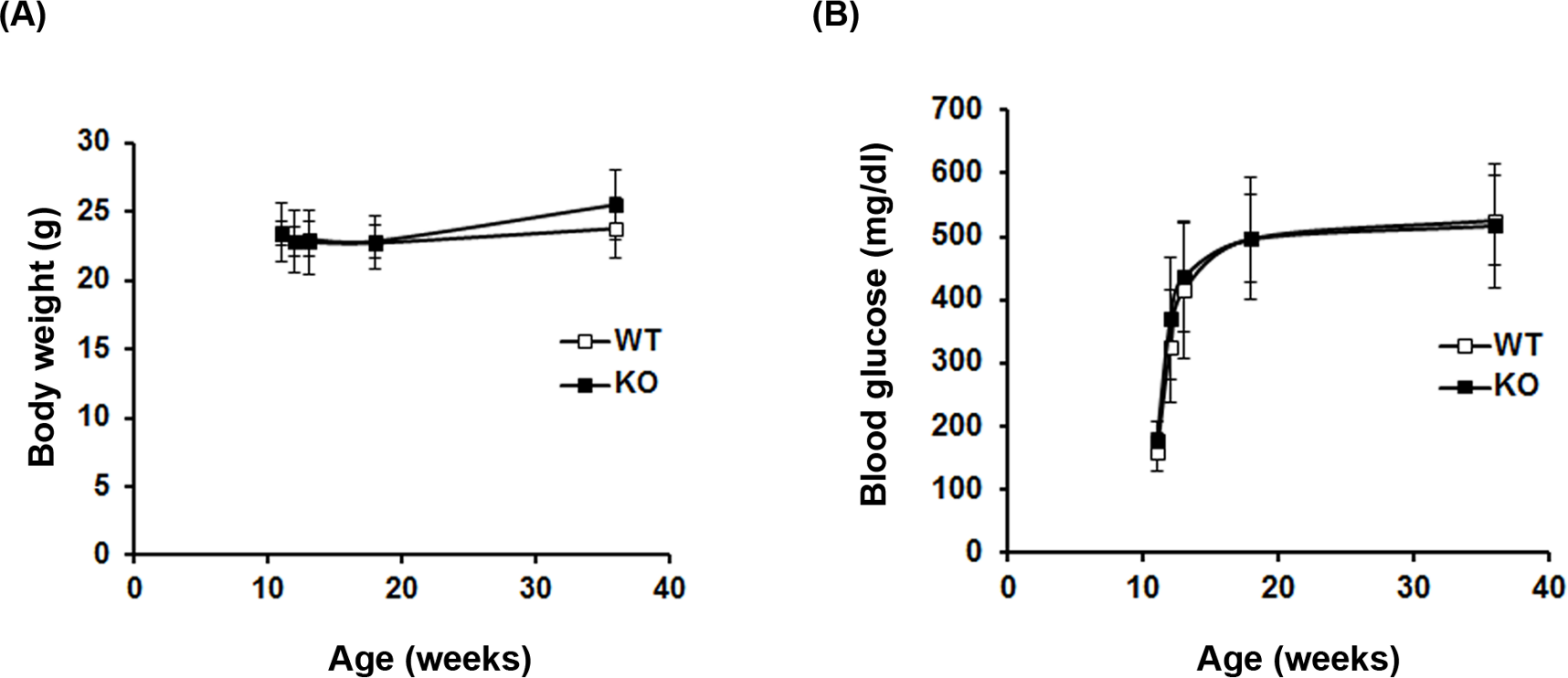
Generation of STZ-induced diabetic mice. Eleven week-old wild type (WT) (n = 10) and SRR-KO (KO) mice (n = 10) were injected with STZ (60 mg/ kg) for 3 consecutive days. Body weight (A) and blood glucose levels (B) were measured for 24 weeks post-STZ treatment.

To establish the DR model in WT and SRR-KO mice, we injected STZ to the mice at the age of 11 weeks and monitored their body weight and blood glucose level. There was no significant difference between WT and SRR-KO mice in body weight and blood glucose level before STZ injection. After the STZ injection, WT and SRR-KO mice gained body weight slightly (Fig 1A). They exhibited an elevation of blood glucose level over 300 mg/dl (our definition of diabetes in this study) 1 week after the STZ injection and maintained high blood glucose levels thereafter (Fig 1B). There was no significant difference in body weight and blood glucose level after STZ injection between WT and SRR-KO mice.

### Expression of SRR in the retina of mice

**Fig 2:**
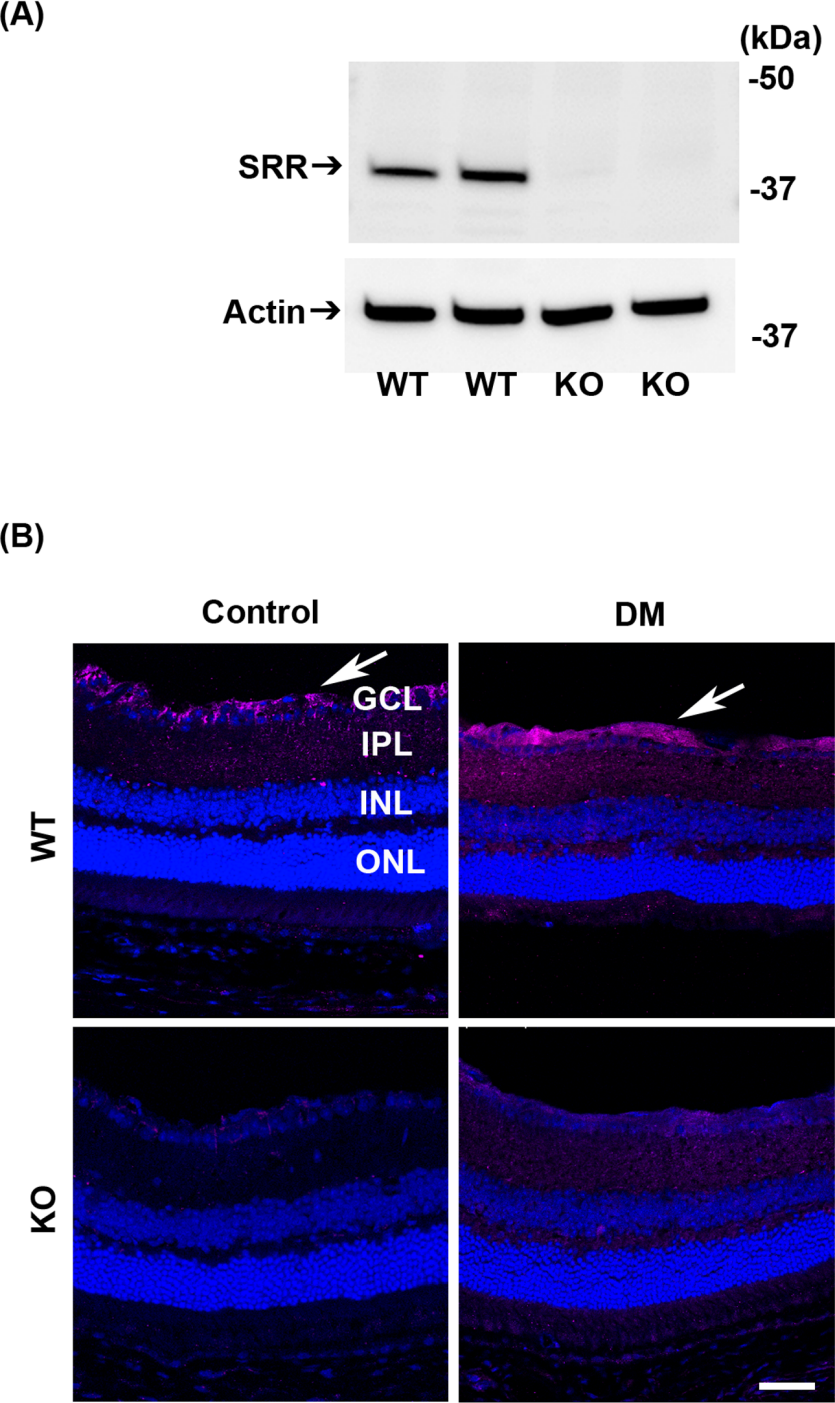
Expression of SRR in mouse retina. (A) Western blot analysis of retina proteins in control (non-treated) WT and KO mice using anti-SRR (upper) and anti-actin (lower) antibodies. The position of protein size markers are indicated on the right side. (B) Immunofluorescence staining of mouse retina from non-treated (Control) and STZ-induced diabetes mellitus (DM) mice using anti-SRR antibody (magenta). Nuclei were counterstained with DRAQ5 (blue). Five retinas per group were examined. Arrows indicate the SRR-immunopositive signals. Scale bar = 50 μm. GCL, ganglion cell layer; IPL, inner plexiform layer; INL, inner nuclear layer; ONL, outer nuclear layer.

We examined the expression of SRR in the retina using western blot analysis and immunohistochemistry (IHC). We detected the band of SRR with expected molecular weight of ~38 kD in WT mice but not in SRR-KO mice (Fig 2A). We examined the localization of SRR in the retina of control and diabetic mice (6 months after STZ injection) using anti-SRR antibody. Immunopositivity of SRR was detected in the GCL of the retina in WT but not in SRR-KO mice. Immunopositivity of SRR was increased in the GCL and IPL in diabetic WT mice (Fig 2B). The specificity of SRR immunopositive signal was evaluated by using SRR-KO mice as negative controls (Fig 2B).

### Attenuation of retinal neuronal cell loss in diabetic SRR-KO mice

**Fig 3:**
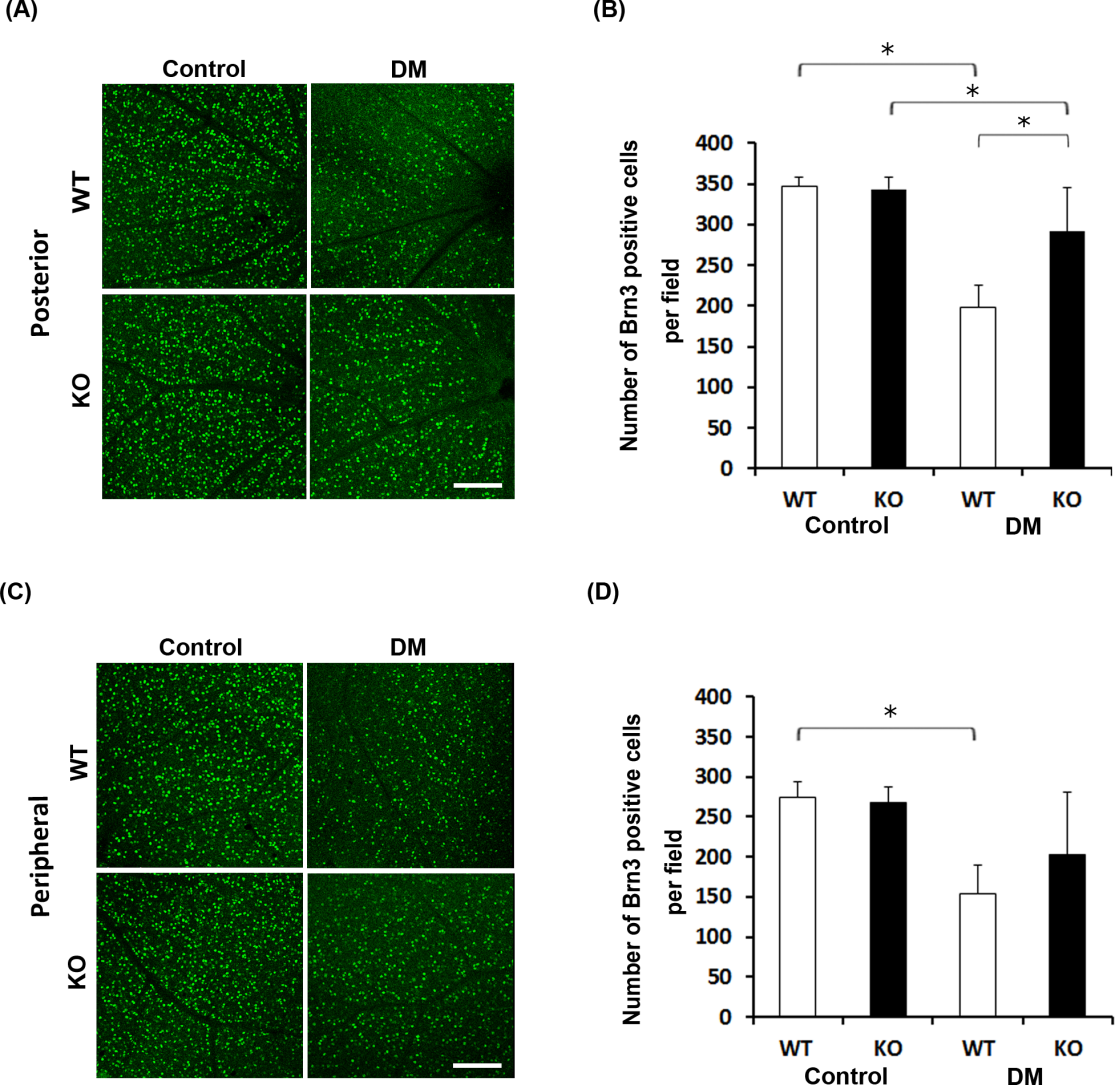
Brn3-immunopositive retinal ganglion cells in non-treated (control) and diabetic (DM) WT and SRR-KO mice. Immunofluorescence staining of retinal ganglion cells in flat-mount retinas using anti-Brn3 antibody. (A) Brn3-labeled retinal ganglion cells in the posterior retina in Control and DM mice. (B) Graph shows the number of Brn3-immunopositive cells in the posterior retina in Control and DM mice. (C) Brn3-labeled retinal ganglion cells in the peripheral retina in Control and DM mice. (D) Graph shows the number of Brn3-immunopositive cells in the peripheral retina in Control and DM mice. We counted the number of Brn3 positive cells in a 0.09 mm^2^ field area. Scale bar = 100 μm. Data are presented as mean ± S. E. M. n = 5 mice per group. *p < 0.05; one-way Analysis of Variance (ANOVA).

To examine the role of SRR in retinal neurodegeneration in diabetic mice, we quantified the number of RGCs expressing Brn3 in WT and SRR-KO mice. There was no significant difference in the number of RGCs between control WT and SRR-KO mice (Fig 3A and 3B). Six months after the onset of diabetes, in the posterior retinas, we found that the number of RGCs expressing Brn3 was significantly lower in diabetic WT and SRR-KO mice than those of control mice (Fig 3A and 3B). The number of RGCs expressing Brn3 of diabetic SRR-KO mice was significantly higher than that of diabetic WT mice (Fig 3A and 3B). In the peripheral retina, the number of RGCs expressing Brn3 was significantly lower in diabetic WT than those of control WT mice (Fig 3C and 3D). There was no significant difference in the number of RGCs expressing Brn3 between diabetic WT and SRR-KO mice (Fig 3C and 3D).

### Reduction of thickness of inner retinal layer is attenuated in diabetic SRR-KO mice

**Fig 4:**
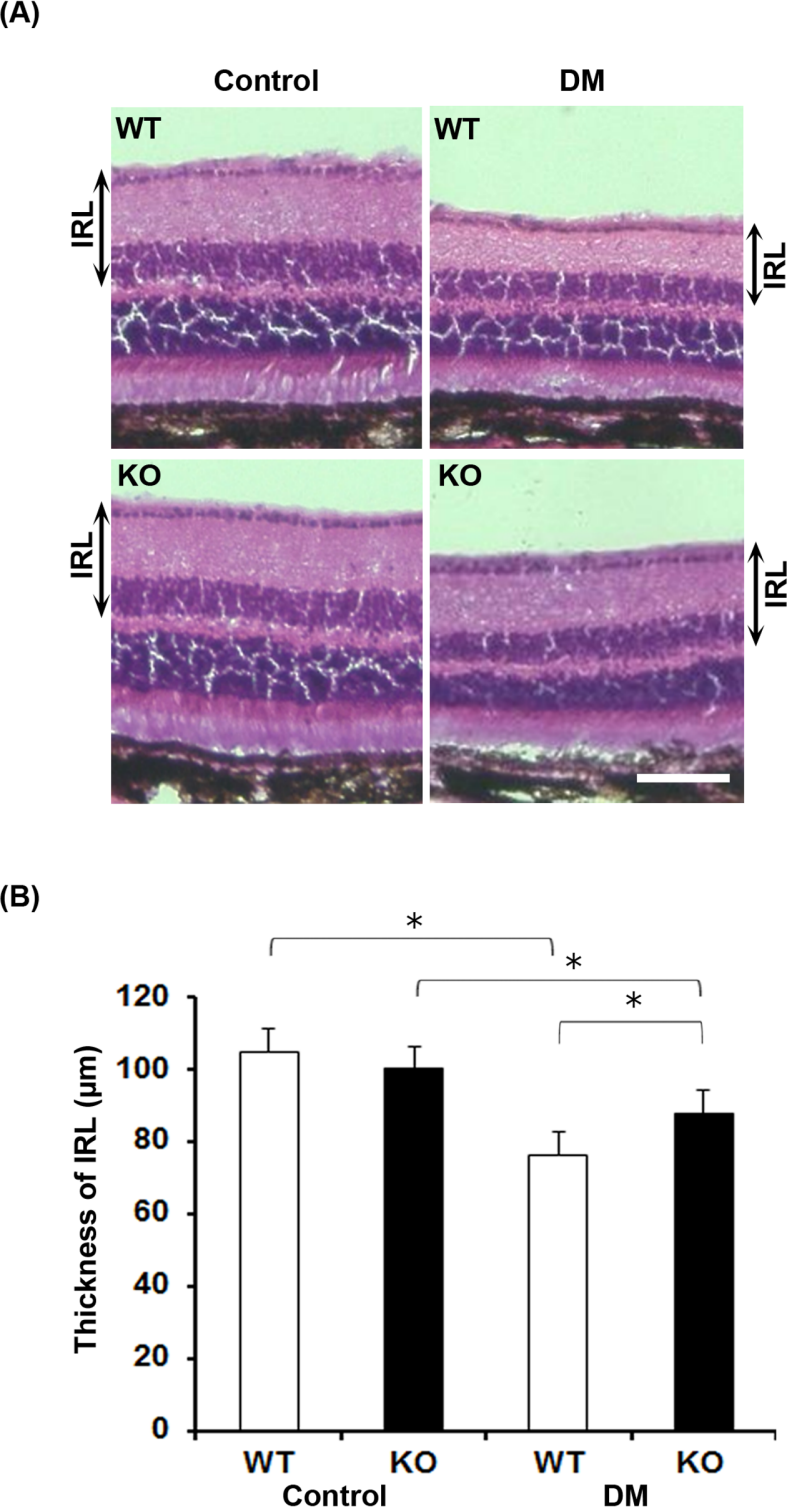
Hematoxylin and eosin (H&E) staining of retinas from non-treated (control) and diabetic (DM) WT and SRR-KO mice. (A) Representative images of H&E staining. (B) Graph shows the thickness of the inner retinal layer (IRL). Scale bar = 100 μm. Data are presented as mean ± S. E. M. n = 5 mice per group. *p < 0.05; one-way ANOVA.

We next analyzed the thickness of inner retinal layer (IRL) with H&E staining. There was no significant difference in the thickness of IRL between control WT and SRR-KO mice (Fig 4A and 4B). Six months after the onset of diabetes, the thickness of IRL in diabetic WT and SRR-KO mice was significantly reduced than that of control mice (Fig 4A and 4B). The reduction of thickness of IRL in diabetic SRR-KO mice was less than that of diabetic WT mice (Fig 4B).

### Reduced number of acellular capillaries in diabetic SRR-KO mice

**Fig 5:**
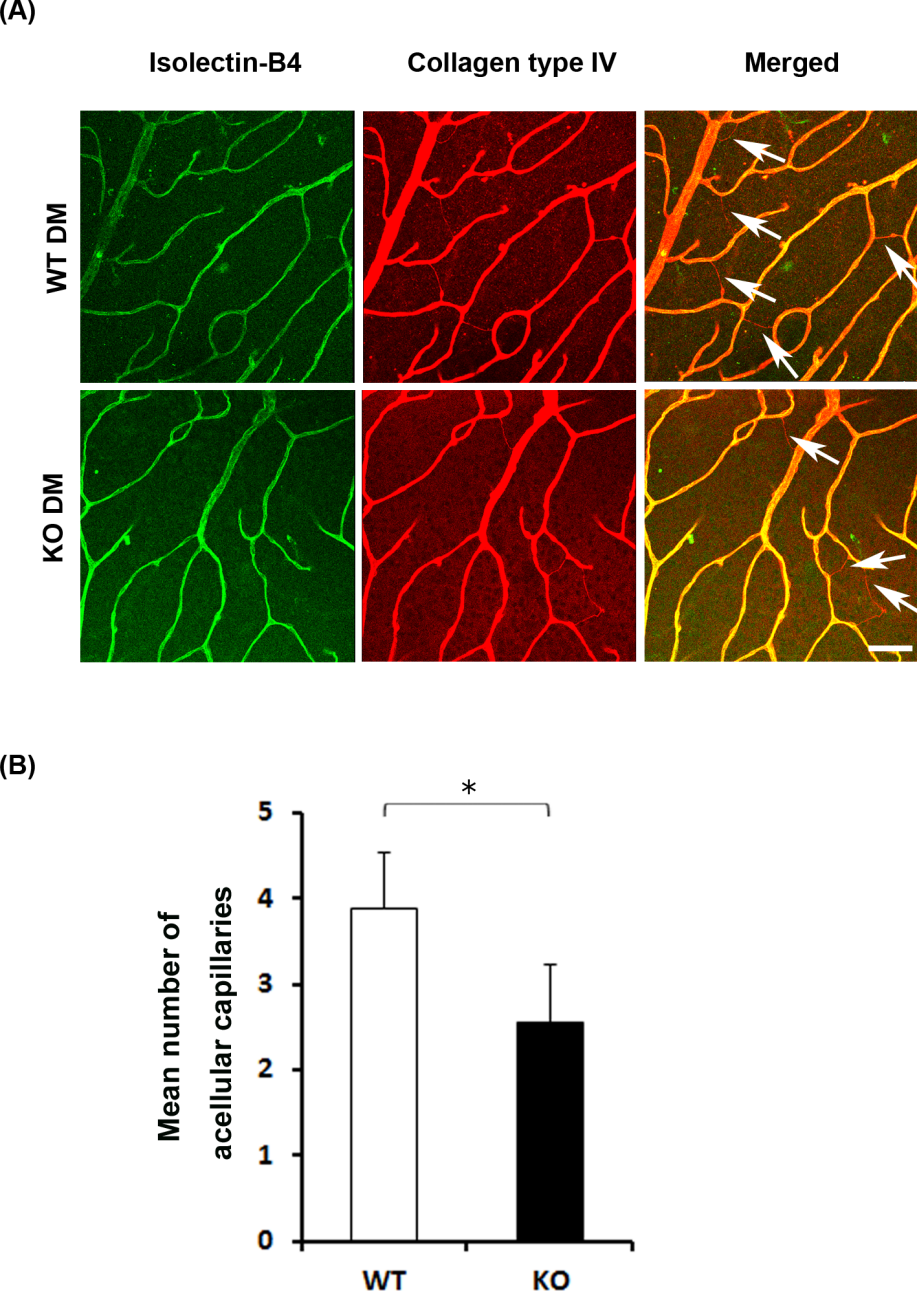
STZ-induced microvascular changes in diabetic (DM) WT and SRR-KO mice. (A) The retinal vasculature were dual-labeled with isolectin-B4 (green) and anti-collagen IV antibodies (red) in flat-mount retinas. Arrows in the merged images indicate collagen IV positive and isolectin negative acellular capillaries. (B) Graph shows mean number of acellular capillaries in diabetic WT and SRR-KO mice. We counted the number of acellular capillaries in a 0.09 mm^2^ field area. Scale bar = 50 μm. Data are presented as mean ± S. E. M. n = 15 fields/mouse, 5 mice per group. *p < 0.05; two-tailed Student’s *t*-test.

Diabetes leads to microvascular damage such as acellular capillary formation. Acellular capillaries have naked basement membrane can be detected as anti-collagen IV antibody-positive and isolectin-B4-negative features [21]. To evaluate the microvascular damage in diabetic WT and SRR-KO mice six months after the onset of diabetes, we counted the number of acellular capillaries (Fig 5A and 5B). We found that the number of acellular capillaries in diabetic SRR-KO mice was significantly smaller than that of diabetic WT mice (Fig 5B).

## Discussion

In the present study, we established STZ-induced diabetic model in WT and SRR-KO mice. Six months after the onset of diabetes, we found that the number of RGCs was higher and the number of acellular capillaries was lower in SRR-KO mice than those of WT mice. Furthermore, the reduction of thickness of IRL in SRR-KO mice was smaller than that of WT mice. These results indicate the degeneration of retinal neuronal cells and microvascular damage induced by diabetes were attenuated in SRR-KO retinas.

We characterized STZ-induced DR of WT and SRR-KO mice 6 months after the onset of diabetes. We found the attenuation of RGC loss in SRR-KO mice, which is in consistent with the previous study that RGCs in WT mice are more vulnerable to acute NMDA toxicity than that of SRR-KO mice [18]. The difference of NMDA-mediated excitotoxicity between WT and SRR-KO mice might attribute to the differential level of D-serine in retinas. In the posterior retina, the number of RGCs expressing Brn3 was significantly lower in diabetic SRR-KO mice than that of control SRR-KO mice, but this difference was not observed in the peripheral retina. This regional different effect of RGC loss might be attributed to the SRR/D-serine independent neurodegeneration mechanism. There is controversy whether or not diabetes induces RGC loss in STZ-induced diabetic C57BL/6 mice [22]. Experimental conditions, such as used dose and protocol for STZ injection, timing of RGC loss evaluation, and methods to detect RGC loss, affect the conclusion of the RGC loss by STZ induced diabetes in C57BL/6 mice. We detected significant RGC loss in our study. Our experimental condition with relative high-dose of STZ, evaluation of RGC loss 6 months after the onset of diabetes, and histological examinations of number of Brn3 immunopositive cells and thickness of inner retinal layer is similar to the experimental conditions detected RGC loss in STZ-induced diabetic C57BL/6 mice [23, 24].

In our study, increased immunopositivity of SRR was found mainly in GCL in diabetic WT mice as reported [17]. The specificity of SRR immunopositive signal was evaluated by using SRR-KO mice as negative controls. As the expression of SRR is enhanced by inflammatory stimuli in vivo [25], increased expression of SRR might be a result from inflammation occurring in diabetes. In our hand, we could not evaluate cellular distribution of SRR signals localized in neurons or glial cells in the retina.

In this study, we demonstrated for the first time that reduction of acellular capillaries formation in SRR-KO mice under long-term diabetic condition. Appearance of acellular capillaries is reported as the damage to the vessels by ischemia due to obliteration of retinal microvessels [2]. The interactions among neurons, glial cells, and vascular cells are important for maintaining the vascular structure in retinas [26]. Retinal neurodegeneration has been found in diabetic retinas without any abnormalities of capillaries, suggesting that retinal neurodegeneration precedes acellular capillary formation [27]. The RGCs express growth factor such as platelet-derived growth factor (PDGF-A), and retinal astrocytes express it’s receptor (PDGFαR) [28]. PDGF-A induces migration and proliferation of retinal astrocytes, which promote the survival of endothelial cells [29]. Thus, the endothelial cells loss is thought to attribute to retinal neurodegeneration, which causes acellular capillaries observed in diabetic WT mice. brain.

In conclusion, we found the retinal neurodegeneration and microvascular damage induced by diabetes was significantly attenuated in SRR-KO mice. Thus, the suppression of SRR activity may have protective effects on the retinal neurons and vasculature in diabetes. Further studies will need to clarify the mechanisms that SRR plays a role in retinal microvascular damage in diabetes.
